# Oil-Based Polymer Coatings on CAN Fertilizer in Oilseed Rape (*Brassica napus* L.) Nutrition

**DOI:** 10.3390/plants10081605

**Published:** 2021-08-05

**Authors:** Petr Škarpa, Dominika Mikušová, Jiří Antošovský, Milan Kučera, Pavel Ryant

**Affiliations:** 1 Faculty of AgriSciences, Mendel University in Brno, Zemědělská 1, 613 00 Brno, Czech Republic; petr.skarpa@mendelu.cz (P.Š.); dominika.mikusova@mendelu.cz (D.M.); jiri.antosovsky@mendelu.cz (J.A.); 2 Research Institute of Chemical Technology (VUCHT a.s.), Nobelova 34, 836 03 Bratislava, Slovakia; mkucera@vucht.sk

**Keywords:** control release fertilizer, yield, oiliness, nitrogen losses, nitrate leaching

## Abstract

Fertilizer coating can increase the efficiency of N fertilizers and reduce their negative impact on the environment. This may be achieved by the utilization of biodegradable natural coating materials instead of polyurethane-based polymers. The aim of this study was to detect the effect of calcium ammonium nitrate (CAN) fertilizer coated with modified conventional polyurethane enhanced with vegetable oils on the yield and quality of *Brassica napus* L. compared to CAN fertilizer with a vegetable oil-based polymer and to assess the risks of nitrogen loss. Three types of treatments were tested for both coated fertilizers: divided application (CAN, coated CAN), a single application of coated CAN, and a single application of CAN with coated CAN (1:2). A single application of coated CAN with both types of coating in the growth stage of the 9th true leaf significantly increased the yield, the thousand seed weight, and oil production compared to the uncoated CAN. The potential of using coated CAN may be seen in a slow nitrogen release ensuring the nitrogen demand for rapeseed plants throughout vegetation and eliminating the risk of its loss. The increased potential of NH_4_^+^ volatilization and NO_3_^−^ leaching were determined using the uncoated CAN fertilizer compared to the coated variants. Oil-based polymer coatings on CAN fertilizer can be considered as an adequate replacement for partially modified conventional polyurethane.

## 1. Introduction

With the world’s exponential population growth and diminishing of arable lands, the agriculture industry has faced a great challenge of crop and food resources for the past decades [1,2]. Predictions are that the earth’s population could approach 9.5 billion by 2050, which may result in an almost double increase in food demand and crop production. In one specific example, cereal production is expected to increase from 940 million tons to 3 billion tons a year [3,4]. Satisfying increasing grain yield demands has been achieved by enhancing the use of mineral fertilizers to cropland soil. However, the excessive application of fertilizers presents one of the main sources of polluting soil (heavy metals), water (nitrates leaching into groundwater), and air environments (emission of greenhouse gases), which could be a threat to human health [5,6].

Nitrogen occupies a unique position among essential plant nutrients. Nitrogen and water availability are considered the two major limiting factors in plant growth and development of metabolic processes—nutrient distribution, photosynthesis, biomass, and ultimately yield building [7,8,9]. The deficiency of nitrogen strongly decreases chlorophyll content, enzymatic activity, photosynthesis, respiration rate, and yield of crops [10]. Nitrogen can be directly absorbed by plant roots in inorganic forms (mineral nitrogen) as ammonium (NH_4_^+^) and nitrate (NO_3_^−^). These forms are the key components of nitrogen fertilizers such as ammonium nitrate (AN) and urea included in the two most widespread nitrogen fertilizers [11]. 

According to FAO, world demand of nitrogen (N), phosphate (P_2_O_5_), and potash (K_2_O) fertilizers were reported to be in total up to 184.0 mil tons in 2015. The forecast for 2022 could be up to 200.9 mil tons (nitrogen fertilizers present up to 111.6 mil tons) [12,13]. Despite the fact that the use of nitrogen fertilizers plays an essential role in meeting the demand for crop production, nitrogen use efficiency (NUE) is relatively low due to their excessive use (in general between 25–50%) and often leads to losses of redundant nitrogen from agroecosystems [14]. Nitrogen losses due to gaseous emissions of ammonia (NH_3_), nitric oxide (NO), nitrous oxide (N_2_O), and dinitrogen (N_2_) along with leaching and runoff in the forms of ammonium (NH_4_^+^), nitrite (NO_2_^−^), and nitrate (NO_3_^−^) present an alarming threat to the environment [15]. 

Leakage prevention of nitrates may present one of the greatest environmental challenges in terms of nitrogen fertilizer use. Nitrogen losses, caused by NO_3_^−^ leaching from soil into water, represent a loss of soil fertility and also pose a threat to the environment and subsequently to human health [5,16]. Increased nitrate levels present in drinking water create a risk of cancer, heart disease, and methemoglobinemia in babies [17]. According to calculations by Grizzetti et al. [17], up to 50% of the European population live in areas with a concentration of nitrates in water exceeding 25 mg·L^−1^, and up to 20% live in areas where nitrates exceed the recommended level of 50 mg·L^−1^. As already mentioned, nitrate coming from agriculture is the most common contaminant in the world’s groundwater aquifers [18]. In the European Union, up to 38% of water bodies are significantly under pressure from agricultural pollution [19].

The application of controlled-release fertilizers (CRFs) is one way to improve nutrient use efficiency, reduce nitrogen loss, and contribute to minimizing environmental pollution, providing a better compromise among soil fertility, yield, and grain quality [20,21,22]. CRFs prove the potential to decrease the fertilizer application rate by 20% or 30% of the recommended value to achieve the same yield [23]. According to Trenkel [23] and Shaviv [24], CRFs can be defined as coated or encapsulated fertilizers by water-insoluble, semipermeable, or impermeable-with-pores materials, for which the factors determining the rate, pattern, and the duration of release have been known and regulated during the fabrication. Coating materials can be divided into two categories—inorganic materials (e.g., sulfur, bentonite, phosphogypsum) and organic polymers consisting of synthetic polymers derived from petroleum-based derivates (polyurethane, polyethylene, alkyd resin, etc.) or natural polymers (e.g., vegetable oil, starch, chitosan, cellulose) [23,25]. One of the most effective methods of preparing CRFs and thus the reduction of nutrient losses is by coating the surface of fertilizer with polyurethane materials. However, these coating materials are commonly linked with high costs and come from non-renewable petrochemical productions [26,27]. Furthermore, studies have shown that the residue of polyurethane shells in soils is difficult to degrade and may cause potential environmental risk [28]. This is one of the reasons why the agricultural industry has been searching for cheap, degradable, and renewable bio-based materials [29]. Vegetable oil is considered to be the most significant material for bio-based polymers, and polymeric material preparation to be an adequate substitution for polyurethanes [30]. Recent studies have shown that the use of oil-based polymers as coating materials led to gradual, uniform nutrient release and proved a high rate of biodegradability [31,32]. The most widely used vegetable oils to produce bio-based polymers are castor, linseed, canola, sunflower, palm, tobacco, corn, soy, and rapeseed [33].

The positive effect of CRFs in rapeseed cultivation has been described in several studies. The data show that the addition of coated N-fertilizers significantly increases the yield and quality of rapeseed [34,35]. Increased yield rates can be a consequence of advanced root volume and the improvement of plant biomass accumulation (especially during the growth stages of stem elongation, flowering, and harvest), extending the photosynthetic lifespan of pods [36,37,38].

The aim of our study was to evaluate the differences in the effectiveness of two newly developed coated fertilizers in nutritional status and yield of rapeseed and to assess their environmental impact (especially nitrate leaching). We assumed that the CAN fertilizer coated with a polymer-based on vegetable oil might provide comparable results with the same fertilizer coated with a modified conventional polyurethane.

## 2. Results and Discussion

The evaluation of the effect of coated fertilizers was created by comparing the data within the groups using the treatments with the same fertilizer application system (divided, single, and blends). Each method of fertilization was assigned with a control treatment (the treatments D and S). D served as the control variant for the group with a divided application, and S served as a control for the group with a single application and blends.

### 2.1. Yield and Oiliness of Rapeseed and N Content in Plant Biomass

**Figure 1:**
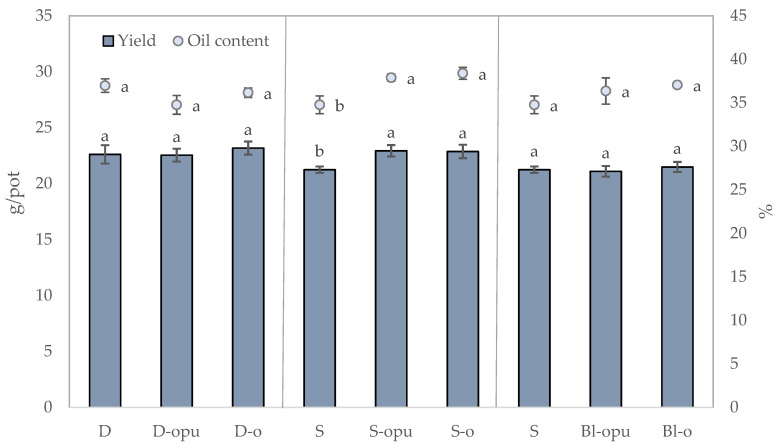
Rates of yield and oiliness of rapeseed. The groups of the treatments D—divided application; S—single application; Bl—blend. The columns represent the mean (*n* = 4), error bars present the mean standard deviation (SD). The same letters at the top of the columns describe no statistically significant differences between the treatments (Fisher’s LSD test, *p* ≤ 0.05). Each group of the treatment (D, S, Bl) was evaluated separately.

The appropriate type of fertilizer and method of fertilization is important for the high yield production of rapeseed. Several studies describe the increase in yield and qualitative parameters of crops after using coated fertilizer application [38,39,40,41]. Our study showed that the use of coated CAN fertilizers has no negative effect on the yield and qualitative parameters of winter rapeseed. Statistical evaluation of the data shown in Figure 1 revealed no significant differences between the treatments in the groups with divided application (D, D-opu, D-o) and blends (S, Bl-opu, Bl-o). A significant positive effect was recorded in the group of treatments with a single application of coated CAN fertilizers (opu-CAN-oil-based polyurethane-coated CAN; o-CAN-oil-based polymer-coated CAN) in seed yields and oil contents. Seed yields of this group showed a trend of opu-CAN > o-CAN > CAN with opu-CAN up to 18% higher in comparison to the uncoated CAN. Similar results were recorded in the study by Tang et al. [42], in which a single basal application of coated nitrogen fertilizers contributed to the increase of the yield and rice quality in comparison to the divided application. A different trend was recorded in the case of the oil content that reached up to 5.5% higher after a single application of oil-coated CAN fertilizer compared to the use of the uncoated CAN fertilizer. The presumption was that the total nitrogen applied in the single application of coated fertilizers was released over a longer period of time and thus was present in the phase of the seed formation confirmed by Tian et al. [38]. In this study, the increase was recorded by an average of 17.3% after the application of coated fertilizers in rapeseed yield rates compared to the control. This study also proved that lower doses of the total N applied in coated fertilizers contributed to a yield increase of 14.2%, which confirmed their environmental potential in terms of nitrogen release. The study by Lu et al. [43] showed the positive effects of CRFs application on rapeseed yield manifested in the increase of rapeseed pods from 27 to 32% in comparison to non-coated urea. In comparison to the treatments with coated CAN fertilizers, a single application of the uncoated CAN (treatment S) proved the decline in the parameters of oil production and thousand seed weight (TSW) shown in Table 1. Similar positive effects of coated CAN fertilizers were proved on yield and qualitative parameters of rapeseed. It can be concluded that o-CAN may be a proper alternative instead of opu-CAN.

**Figure 2:**
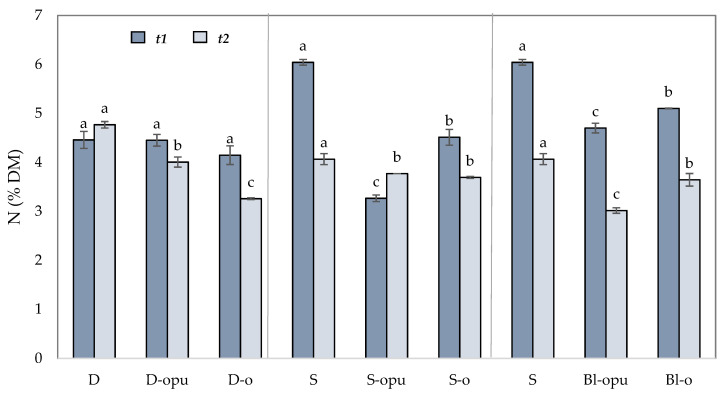
Nitrogen concentration (% DM) in aboveground plants dry matter collected in two growth stages *t1* and *t2* of rapeseed. The groups of treatments D—divided application, S—single application; Bl—blend; opu—oil-based polyurethane polymer, o—oil-based polymer. The columns represent the mean (*n* = 4), error bars present the mean standard deviation (SD). The same letters at the top of the columns describe no statistically significant differences between the treatments (Fisher’s LSD test, *p* ≤ 0.05). Each group of the treatment (D, S, Bl) was evaluated separately.

The data from Figure 2 indicate a connection between the yield rates and the nitrogen concentration in aboveground plant biomass. In general, plants can only consume a part of nutrients (in our case nitrogen) from conventional fertilizers, and the rest may be subject to losses to the environment [44]. This trend is mainly visible in the treatment with the application of conventional uncoated CAN fertilizer in a single dose (S), resulting in a significantly lower concentration of nitrogen in plant biomass in the growth stage of flower bud emergence (*t2*) compared to the growth stage of stem elongation (*t1*). This decrease indicates that the overdose of quickly released nitrogen in uncoated CAN fertilizer led to N-loss available for direct plant consumption and ultimately caused the lowest yield and oil content. The declining trend in the supply of the available form of N, released from conventional uncoated CAN, during the period and the increased supply of mineral N released from coated CAN is also evident from the assessment of N content in aboveground biomass (Table 2). The nitrogen content in the plant shows a gradual release of the available forms of this nutrient from the coated CAN that is particularly evident in the group of singly applied fertilizers (S). While the nitrogen content detected in the aboveground mass of rapeseed fertilized with uncoated CAN (S) was detected almost 4 and 2 times higher in the term *t1* compared to the treatments with coated CAN (S-opu, S-o) in the term *t2,* the nitrogen content of the treatments fertilized with coated fertilizers was increased. These values show that the oil-coated CAN is able to release nitrogen more rapidly than the oil-based, polyurethane-coated CAN and thus may supply the plant’s demand for this nutrient. Nitrogen contents in plants, treated with coated fertilizers applied in blends with conventional CAN (Bl-opu, Bl-o), can confirm this trend.

The relationship between the optimal nitrogen supply and its impact on the yield and oil content of rapeseed is described in many studies [45,46]. A similar trend was recorded in the treatments with coated CAN fertilizers applied in blends with the uncoated CAN fertilizer (Bl-opu, Bl-o). Nitrogen content in plant biomass in the growth stage of stem elongation decreased about 1.3% and 0.9% compared to the uncoated CAN fertilizer applied in a single dose. The N content in plant rapeseed showed the most even N pumping during vegetation in the variant with divided application and a single application of coated CAN fertilizers.

### 2.2. Mineral Nitrogen Content in the Soil 

The release of nitrogen from coated CAN fertilizers significantly affected the dynamic change of the soil mineral N (N_min_) content in the growth process of rapeseed. Contents of N_min_ and its ionic forms (NO_3_^−^, NH_4_^+^) were determined in the soil in three experimental phases (*t1*–*t3*,). Although, enough of the available nitrogen can be essential for direct plant consumption. The excessive content may inevitably increase its loss in soil [47]. Average contents of N_min_ in soil (without differencing into layers), shown in Table 3, serve as an overview of nitrogen release development in the treatments during the rapeseed vegetation. 

**Figure 3:**
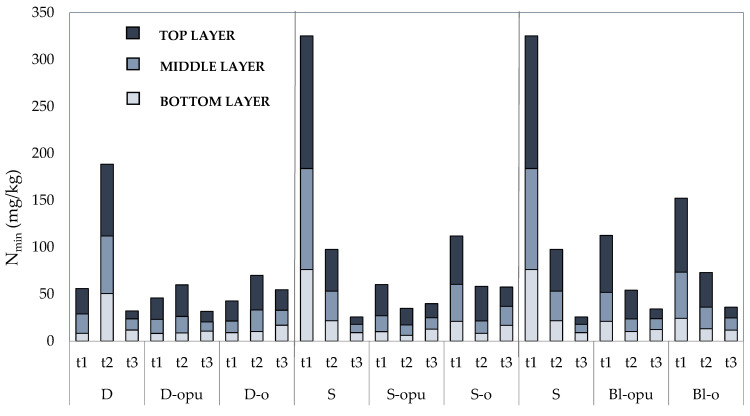
Contents of mineral nitrogen (N_min_) in soil (top, middle and bottom layer of pot) in three experimental phases (*t1, t2, t3*). Groups of treatments D—divided application, S—single application; Bl—blend; opu—oil-based polyurethane polymer, o—oil-based polymer. The columns represent the mean (*n* = 4).

One of the important aspects of coated fertilizers is the longevity of nutrient release in sufficient levels for plant uptake. The use of coated CAN fertilizers in each form of the application (D, S, and Bl) has shown a positive effect on N_min_ release pattern, as can be seen from Figure 3. The effect was visible, especially in the period between the first (*t1*) and the second term (*t2*) of soil samples collection that was significantly milder compared to conventional uncoated CAN.

The relatively accelerated release of nitrogen was observed in high N_min_ concentration after the application of fertilizers (*t1* single application, *t2* divided application) in the treatments with conventional uncoated CAN shown in Table 3. Rapid release N_min_ was visible mainly in the single application in which N_min_ concentration decreased rapidly up to 65.4% between *t1* and *t2* (up to 22 days). Our assumption was that although the part of the soil N_min_ was obtained from the soil through plant roots, the great contrast in N_min_ concentration was due to N loss (NH_4_^+^ volatilization and NO_3_^−^ leaching) between *t1* and *t2*. On the contrary, the data of the soil samples, collected in the harvest time (*t3*), showed relatively high levels of N_min_ in the treatments with divided (especially D-o) and single (S-opu, S-o) application of fertilizers in comparison with conventional CAN treatments. Dynamic of gradual N_min_ release was most visible after a single application of both coated CAN (S-opu, S-o) with no definite decrease in N_min_ content in *t3*. A single application of oil-based polyurethane-coated CAN fertilizer (S-opu) caused an increase by 14.2% in *t3* in mineral nitrogen content compared to *t2* in soil. These findings corresponded to the data of yield and qualitative parameters (Figure 1), in which a single application of coated CAN fertilizer (S-opu) proved to be the most effective. The assumption was that the amount of released nitrogen reached sufficient levels for the plant demand in the time of the experiment duration from these treatments, thus leading to the increased nitrogen use efficiency and subsequently to a more positive environmental impact (lower risk of N loss). Our data are consistent with the findings of Xiao et al. [48], who described that the total N_min_ content continued gradually to an increase in the top layer of soil on the ninetieth day after the application of coated fertilizers, while high levels were maintained in the middle and bottom layer of soil.

The positive effect of coated CAN fertilizers on N_min_ content was also visible in the nitrogen distribution between soil layers during the experiment (Figure 3). The application of conventional uncoated CAN fertilizer (D and S treatment) showed high N_min_ concentrations mainly in the top and middle layers of the soil right after fertilization. The treatments with coated CAN fertilizers showed that N_min_ content was, in general, focused mainly on the top layer of the soil during *t1* and *t2.* N_min_ content was evenly distributed between each layer of the soil in the harvest time (*t3*). This indicates that both coated CAN fertilizers (opu-CAN and o-CAN) proved a high ability of gradual nitrogen release leading to more efficient nitrogen use by the plant and a reduction in the environmental risk. A gradual N_min_ release by coated fertilizers was also described in the study by Zheng et al. [49], who found that the application of coated fertilizers resulted in enhanced N_min_ concentration in soil, especially during later crop stages. 

Considering the placement of the fertilizers (the placement on the soil surface without incorporation to the soil), the highest potential for the NH_4_^+^ volatilization is most likely to be closest to the soil surface [50]. Ammonium nitrate (used CAN in our experiment), depending on N dose and irrigation, belongs to the conventional nitrogen fertilizers with a high potential of NH_4_^+^ volatilization [51]. 

**Figure 4:**
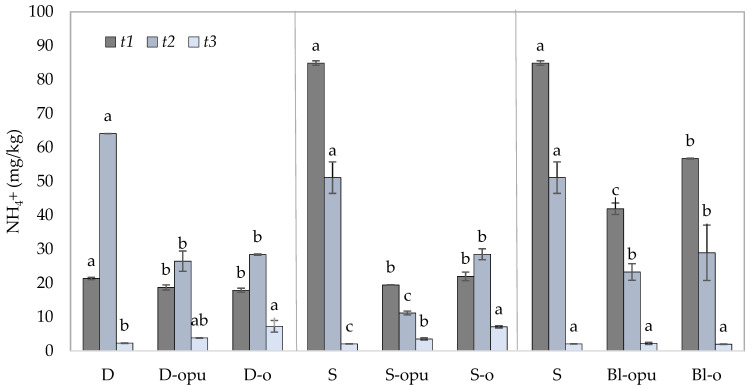
Contents of NH_4_^+^ in the top layer of the soil samples collected in three experimental phases (*t1*–*t3*). Groups of treatments D—divided application, S—single application; Bl—blend; opu—oil-based polyurethane polymer, o—oil-based polymer. The columns represent the mean (*n* = 4), error bars present the mean standard deviation (SD). The same letters at the top of the columns depict no statistically significant differences between the treatments (Fisher’s LSD test, *p* ≤ 0.05). Each group of the treatment (D, S, Bl) was evaluated separately.

This assumption was confirmed by the data obtained from the top layer of the soil samples (Figure 4). The data showed the greatest potential for NH_4_^+^ volatilization in the treatments with conventional uncoated CAN (D and S treatments) expressed in significantly high NH_4_^+^ concentrations in *t1* and *t2*. Analogous to N_min_, the uncoated CAN potential of volatilization was visible between *t1* and *t2,* in which the NH_4_^+^ concentration decreased up to 39.8% in soil. Higher NH_4_^+^ concentrations were accountable to the use of conventional uncoated CAN (1/3 of the total N dose) after the application of blend fertilizers (Bl-opu, Bl-o). Similarly, the S variant (a single application of uncoated CAN) was resolved in its rapid release. NH_4_^+^ contents in Bl-opu and Bl-o were detected almost over half lower in *t1* than in the S treatment; therefore, major risks of NH_4_^+^ losses were not found. In addition to the volatilization, a rapid NH_4_^+^ release also presents the risk of the increased concentration of nitrates as an initial component of nitrification in soil and thus increased the risk of NO_3_^−^ leaching [52]. 

The positive effect of coated fertilizers was expressed by significantly lower NH_4_^+^ concentrations during *t1*–*t3* in comparison to conventional uncoated CAN. The data were indirectly consistent with the findings of Xiao et al. [48], who mentioned that the application of coated fertilizers resulted in lower NH_4_^+^ rates in soil samples in comparison to conventional uncoated nitrogen fertilizer. A gradual NH_4_^+^ release was also expressed by the increase of NH_4_^+^ concentration in the top layer of the soil in *t2*. This fact was noticeable in the treatments of D-opu (up to 41.3%), D-o (up to 58.8%), and S-o (up to 29.7%). The treatments with a divided and single application of fertilizers were proved to be the most efficient in terms of the longevity of NH_4_^+^ release. These types of fertilizer applications showed significantly higher NH_4_^+^ contents in *t3* treatments compared to the treatments with conventional uncoated CAN. On the contrary, NH_4_^+^ contents showed no significant difference in the S treatment in Bl-opu and Bl-o. This led to an assumption that all nitrogen contained in coated fertilizers and applied in blends was released during the rapeseed vegetation, predetermining the blend application as the most suitable alternative.

**Figure 5:**
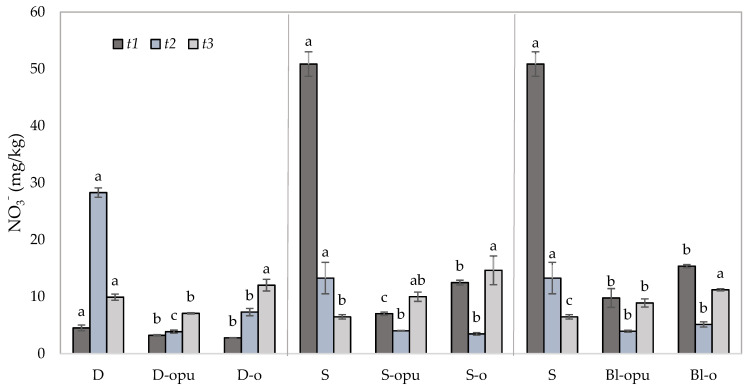
Contents of NO_3_^−^ in the middle layer of soil samples collected in three experimental phases (*t1*–*t3*). Groups of treatments D—divided application, S—single application; Bl—blend; opu—oil-based polyurethane polymer, o—oil-based polymer. The columns represent the mean (*n* = 4), error bars present the mean standard deviation (SD). The same letters at the top of the columns depict no statistically significant differences between the treatments (Fisher’s LSD test, *p* ≤ 0.05). Each group of the treatment (D, S, Bl) was evaluated separately.

**Figure 6:**
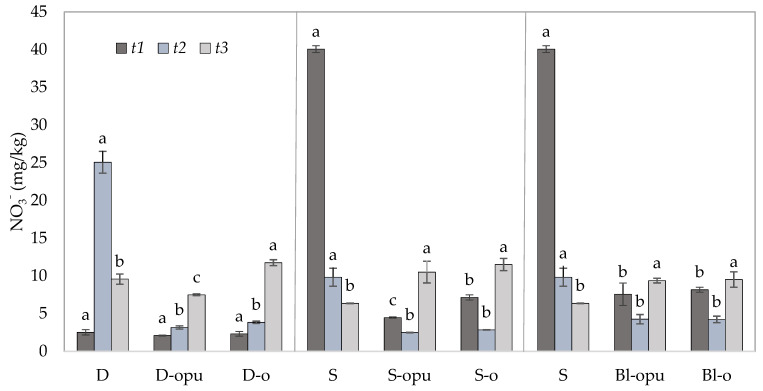
Contents of NO_3_^−^ in the bottom layer of soil samples collected in three experimental phases (*t1*–*t3*). Groups of treatments D—divided application, S—single application; Bl—blend; opu—oil-based polyurethane polymer, o—oil-based polymer. The columns represent the mean (*n* = 4), error bars present the mean standard deviation (SD). The same letters at the top of the columns depict no statistically significant differences between the treatments (Fisher’s LSD test, *p* ≤ 0.05). Each group of the treatment (D, S, Bl) was evaluated separately.

Contents of NO_3_^−^ were monitored as the main potential source of N loss in the soil samples due to their high leaching ability. One of the first studies by Liegel and Walsh from 1976 [53] proved that the application of controlled-release N fertilizers was the most effective technique in sandy irrigated soils with a high risk of nitrate leaching. Preventing the leaching of nitrates presents one of the greatest environmental challenges in terms of nitrogen fertilizer use. The estimation of the potential for N losses due to the NO_3_^−^ leaching from the experimental treatments were provided by the isolation of the data from the bottom and middle layers of soil. The data obtained from the middle layer (ML) of the soil (Figure 5) served for the evaluation of potential NO_3_^−^ migration to the lower layers of the soil, which might consequently lead to its leaching into the groundwater. The data obtained from the bottom layer (BL) of the soil (Figure 6) served to evaluate the potential of nitrates leaching to the groundwater during the rapeseed vegetation and directly after its harvest.

As predicted, significantly, the highest potential for NO_3_^−^ leaching was due to rapid nitrogen release from conventional uncoated CAN fertilizers recorded in single or divided CAN application. The potential for NO_3_^−^ leaching after uncoated CAN application was possible to confirm from the data of NO_3_^−^ concentrations in *t1* and *t2* shown in Figure 5 and Figure 6. The NO_3_^−^ content of ML and BL was detected over three times higher (>3.3) in the treatment fertilized with a single application of uncoated CAN in *t1* compared to the treatments with coated CAN fertilizers. The data showed that the NO_3_^−^ decrease was found up to 73.9% in ML and up to 75.5% in BL in the S treatment between *t1* and *t2*. Considering the amount and duration (up to 14 days), it is most likely that nitrates of the uncoated CAN fertilizer were lost due to the nitrate leaching. These findings corresponded with the data by Zhang et al. [54], who discovered that the rates of the leached nitrates in water samples were detected significantly higher in comparison to coated urea in the treatments with conventional urea. 

Identical to N_min_ and NH_4_^+^, the positive effect of coated CAN fertilizers was recorded in the form of gradual NO_3_^−^ release over the course of the whole experiment. Gradual release of nitrates was discovered to be the most visible between *t2* and *t3* in coated fertilizers. The increased NO_3_^−^ contents were observed up to 64.7% in ML and up to 119.9% in BL. While the NO_3_^−^ amount was decreased in ML and BL in the treatments fertilized with uncoated CAN, the coated CAN fertilizers were able to supply the plants with nitrogen even in the later stages of the development. Compared to the low levels of NO_3_^−^ content in the treatments with conventional CAN fertilizers (due to rapid nitrogen release and subsequent N loss). This increase correlated with the data of seed yield and qualitative parameters (Figure 1) and can be used as a potential supply of available nitrogen for the next crops. The data correlated with the findings of Xiao et al. [48]. Similar N_min_ release (especially NO_3_^−^) was proved using oil-based polymer-coated CAN, which can be a proper alternative for oil-based polyurethane-coated CAN. This fact is not suitable for future use due to polyurethane’s lower biodegradability. The positive effect of coated fertilizers on nitrates leaching was recorded in several studies [55,56,57,58].

## 3. Materials and Methods

The pot experiment was performed under controlled conditions in the vegetation hall of Mendel University in Brno, Brno, Czech Republic (49°12′36.94″ N and 16°36′49.95″ E). 

### 3.1. Plant Material and Growth Conditions 

Rapeseed (*Brassica napus* subs. *napus*) cv. DK Exception (Bayer s.r.o, Prague, Czech Republic) was used in this study. Mitscherlich pots (STOMA GmbH, Siegburg, Germany) were filled with 6 kg of air-dried and <2 cm sieved soil and placed in the vegetation hall. The properties of the used soil for the pot experiment are shown in Table 4. Ten seeds of rapeseed were sown in 2 cm depth in each pot. Three weeks after sowing, the number of rapeseed plants was adjusted to three plants per pot. 

### 3.2. Experimental Design

In the experiment, coated CAN fertilizers were compared with a conventional uncoated CAN. The same total dose of nitrogen was applied in all treatments using different N sources such as calcium ammonium nitrate (CAN, up to 13% N-NH_4_^+^ and 13% N-NO_3_^−^, Lovochemie a.s., Lovosice, the Czech Republic), oil-based polyurethane-coated CAN (opu-CAN) and oil-based polymer-coated CAN (o-CAN). Coated fertilizers were prepared by spreading the coating on conventional fertilizer CAN using the LDP-3 fluidized bed granulating machine (Changzhou Jiafa Granulating Drying Equipment Co., Ltd., Changzhou, China). The coating consisted of oil-based polyurethane polymer (opu-CAN—coating up to 7.6 wt.%, up to 13% N-NH_4_^+^ and 13% N-NO_3_^−^, VUCHT a.s., Bratislava, Slovakia) and oil-based polymer (o-CAN—coating up to 6.1 wt.%—triglycerides of fatty acids, up to 75 wt.% of which unsaturated were up to 45 wt.%, polylactic acid up to 10 wt.%, up to 13% N-NH_4_^+^ and 13% N-NO_3_^−^, VUCHT a.s., Bratislava, Slovakia). The composition of the polyurethane-based coating (opu-CAN) differed from the conventional polyurethanes prepared by the reaction of the diisocyanates with the polymeric diols. The polymeric diols were replaced with the vegetable oil having hydroxy groups in its structure. The prepolymer, obtained by this way, was finally applied in the crosslinking. These modifications led to a substantial increase in the biodegradable fraction of the coating. The prepolymer was completely replaced with a more biodegradable component in the oil-based coating (o-CAN). The biodegradable fraction of the coating material is further increased in this way. 

**Figure 7:**
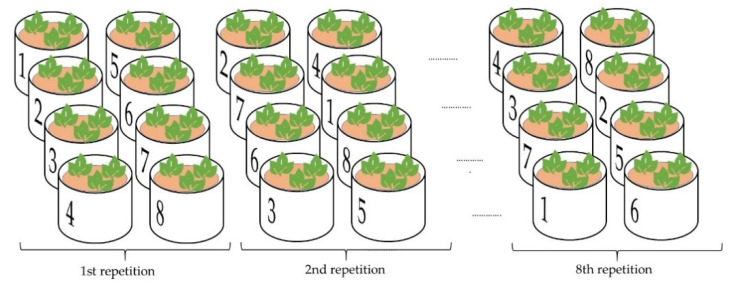
Schematic illustration of the layout of the pots (and their repetition) in the vegetation experiment. Treatment D (1), D-opu (2), D-o (3), S (4), S-opu (5), S-o (6), Bl-opu (7), Bl-o (8).

The individual treatments and fertilizer addition are detailed in Table 5. The fertilizers were applied to the soil surface. Each treatment was replicated 8 times in a complete randomized block design in the vegetation hall (Figure 7). 

The treatments of fertilizer were divided into 3 groups according to the term of application and the type of fertilizer chosen. The first group was the divided application of fertilizers (the designation of the treatments with D). The total nitrogen dose was divided into two parts; the first was applied by the conventional uncoated CAN in the 1st term (1st Fertilization), the second dose was applied by uncoated CAN (treatment D) and coated CAN (treatments D-opu and D-o) in 2nd term (2nd Fertilization). The second and third groups consisted of treatments with a single application of total nitrogen dose in one term (1st Fertilization), where fertilizers of one type (the designation of the treatments with S) and fertilizers of a mixture (the designation of the treatments with Bl) were applied. The fertilizer mixtures (Bl) were created by mixing conventional CAN and coated CAN in a 1:2 ratio (converted to N rate).

**Figure 8:**
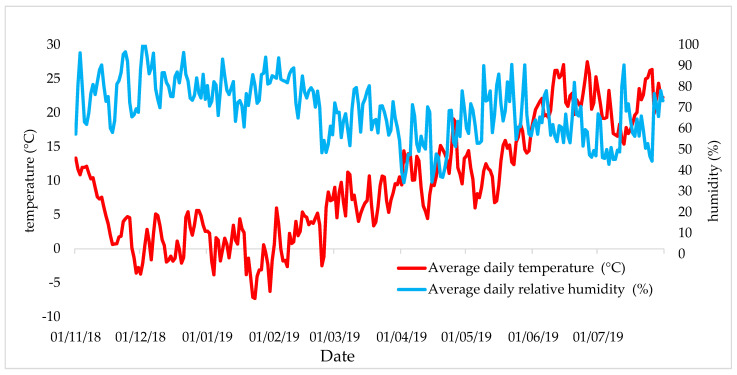
The average daily temperature (°C) and relative humidity (%) in the vegetation hall during the experiment.

The pot experiment was carried out under semi-natural conditions (under a rain shelter) in the vegetative hall. Figure 8 shows the average daily temperature and the average daily relative humidity during the experiment. A controlled watering regime, used identical for all treatments (pots), was in the experiment. Plants were watered to 70% of maximum water holding capacity throughout the growing season. The pots were hand-watered with demineralized water on the soil surface.

Rapeseed plants were harvested manually by cutting above the soil surface from each pot. The rapeseed was threshed using a laboratory thresher (HALDRUP LT-20, Haldrup GmbH, Ilshofen, Germany). 

The rape seeds were purified from coarse impurities by repetitive sifting. Rapeseed yield was measured in three plants within each pot, and the value was adjusted to 9% of moisture. Seed yield was determined by weighing (laboratory scale PCB Kern, KERN & Sohn GmbH, Balingen, Germany) and exceeded as gram per pot (g/pot). Seeds were then counted and hand-ground in mortar for further analysis of the oil content.

### 3.3. Plants and Soil Sampling

**Figure 9:**
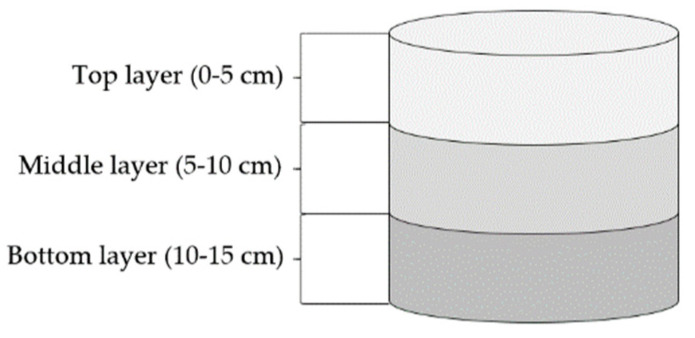
Illustration of soil layers layout in the pot used to determine N_min_ content.

The evaluation of soil mineral nitrogen content (NO_3_^−^, NH_4_^+^) and nutritional plant properties was provided in the soil samples and plant biomass collected in the specific experimental phases shown in Table 6. The collection of the soil samples was carried out by a probe with the aligned tip. After the collection, the soil profile was divided in three zones for the observation of mineral nitrogen movement in soil and subsequently frozen for further analysis (Figure 9). 

The plant biomass was dried at 50 °C and homogenized to determine the nitrogen content in the dry matter.

### 3.4. Analytical Methods

The N_min_ determination was provided according to the methodology by Zbíral et al. [62], who described that nitrate and ammonium nitrogen was extracted from the soils with a solution of neutral salt (1% of K_2_SO_4_). The NH_4_^+^ determination was carried out spectrophotometrically (λ_660 nm_). The NO_3_^−^ contents were determined by ISE (Ion Selective Electrode) [63].

The nitrogen determination was provided in aboveground plant biomass according to the methodology by Zbíral et al. [64]. Nitrogen contents were determined by the Kjeldahl method using the Kjeltec 2300 device (Foss, Hillerød, Denmark).

The thousand seed weight (TSW) determination was performed using a laboratory counter MK (MEZOS spol. s r.o., Hradec Králové, the Czech Republic). The determination was carried out by weighing the number of 2 × 500 seeds to prevent possible measurement errors.

The determination of seed oil content was provided according to the methodology of the Central Institute for Supervising and Testing in Agriculture [65]. The oil content was determined gravimetrically after the extraction of the samples with diethyl ether using the Soxhlet method based on the NMR extraction of rapeseeds in a continuous flow extractor Minispec mq series TD-NMR (Bruker Corporation, Ettlinger, Germany).

### 3.5. Statistical Analysis

The effect of the treatment on the evaluated parameters was statistically analyzed in the STATISTICA 12 program (TIBCO Software, San Jose, CA, USA) [66]. The effect of the treatment on the seed yield, oiliness, oil production, thousand seed weight, nitrogen concentration and content in aboveground plant biomass and the content of mineral nitrogen (ammonium and nitrate) in soil were analyzed separately for each group of the treatment (divided, single and blend application of fertilizers). The normality and homogeneity of variances were verified, respectively, by Shapiro-Wilk and Levene values at *p* ≤ 0.05. The influence of the monitored factors was analyzed via analysis of variance (level of significance *p* ≤ 0.05). The effect of the treatment on the mentioned parameters was analyzed using two-way analyses of variance with the treatment such as fixed effect and the pot used as the random effect to take into account the grouping of individuals in the same pot. The differences between the means were evaluated by the Fisher’s (*LSD*) test.

## 4. Conclusions

The use of coated CAN fertilizer proves the potential to gradually release acceptable nitrogen during the growing season in winter rape nutrition and thus continuously meet the needs of plants. Compared to the effect of conventional CAN, the use of coated CAN fertilizers has been shown to increase the efficiency of nitrogen fertilization and reduce its losses. A suitable method seems to be the application of a mixture of conventional CAN and coated CAN in a ratio of 1:2 during spring fertilization, ensuring a sufficient amount of rapidly releasing N during the regeneration of rapeseed and its slower release during further developmental stages. The CAN fertilizer coated with a biodegradable oil-based polymer proves the ability to release the optimum amount of nitrogen for canola nutrition. The use does not pose a risk of rapid release of mineral N in quantities potentially polluting the atmosphere (ammonia volatilization) and hydrosphere (nitrate leaching). According to these results, the CAN fertilizers coated with a polymer-based on vegetable oils could be used as a replacement for commonly used synthetic polymers based on polyurethane confirming the initial hypothesis.

## Figures and Tables

**Table 1:** Qualitative characteristics of rapeseeds.

Treatment	Oil Production (g/pot)	TSW (g)
D	8.4 ^a^ ± 0.8	30.2 ^a^ ± 2.2
D-opu	7.8 ^a^ ± 0.6	30.1 ^a^ ± 1.5
D-o	8.4 ^a^ ± 0.5	30.9 ^a^ ± 1.6
S	7.0 ^b^ ± 0.5	25.9 ^b^ ± 0.8
S-opu	8.7 ^a^ ± 0.5	30.6 ^a^ ± 1.4
S-o	8.8 ^a^ ± 0.6	30.5 ^a^ ± 1.6
S	7.0 ^a^ ± 0.5	25.9 ^a^ ± 0.8
Bl-opu	7.7 ^a^ ± 0.5	28.1 ^a^ ± 1.3
Bl-o	8.0 ^a^ ± 0.4	28.7 ^a^ ± 1.2

Groups of treatments D—divided application, S—single application; Bl—blend; opu—oil-based polyurethane polymer, o—oil-based polymer, TSW—thousand seed weight. The same letters next to the numbers depict no statistically significant differences between the treatments (Fisher’s LSD test, *p* ≤ 0.05). Each group of the treatment (divided, single, blend) was evaluated separately. The values represent the mean (*n* = 4) ± standard deviation (SD).

**Table 2:** Content of nitrogen in aboveground plant dry matter (mg/plant).

Treatment	Nitrogen Content (mg/plant)	
	*t1*	*t2*
D	68.3 ^a^ ± 0.9	187.4 ^a^ ± 29.2
D-opu	69.6 ^a^ ± 1.4	155.0 ^ab^ ± 2.2
D-o	64.1 ^b^ ± 2.8	123.5 ^b^ ± 8.0
S	135.0 ^a^ ± 1.3	215.6 ^a^ ± 23.9
S-opu	36.9 ^c^ ± 0.2	112.0 ^c^ ± 15.8
S-o	68.6 ^b^ ± 1.6	174.6 ^b^ ± 0.3
S	135.0 ^a^ ± 1.3	215.6 ^a^ ± 23.9
Bl-opu	86.3 ^c^ ± 1.2	135.6 ^b^ ± 3.8
Bl-o	97.4 ^b^ ± 2.8	183.6 ^a^ ± 16.1

Groups of treatments D—divided application, S—single application; Bl—blend; opu—oil-based polyurethane polymer, o—oil-based polymer. The same letters next to the numbers depict no statistically significant differences between the treatments (Fisher’s LSD test, *p* ≤ 0.05). Each group of the treatment (divided, single, blend) was evaluated separately. The values represent the mean (*n* = 4) ± standard deviation (SD).

**Table 3:** Contents of mineral nitrogen (N_min_) in soil (mg/kg) on selected experimental phases (*t1–t3*).

Treatment	*t1*	*t2*	*t3*
D	18.64 ^b^ ± 1.34	62.84 ^b^ ± 1.27	10.69 ^a^ ± 1.04
D-opu	15.31 ^a^ ± 1.22	20.34 ^a^ ± 3.53	10.51 ^a^ ± 0.03
D-o	14.23 ^a^ ± 1.72	23.33 ^a^ ± 1.25	18.21 ^b^ ± 3.09
S	108.42 ^c^ ± 3.37	37.54 ^c^ ± 6.44	8.54 ^a^ ± 0.60
S-opu	20.07 ^a^ ± 0.56	11.62 ^a^ ± 0.56	13.27 ^b^ ± 2.34
S-o	35.33 ^b^ ± 2.89	19.42 ^b^ ± 1.22	19.17 ^c^ ± 2.90
S	108.42 ^c^ ± 3.37	37.54 ^b^ ± 6.44	8.54 ^a^ ± 0.60
Bl-opu	37.51 ^a^ ± 6.01	18.05 ^a^ ± 3.02	11.31 ^b^ ± 1.17
Bl-o	50.73 ^b^ ± 2.22	24.34 ^ab^ ± 5.09	11.99 ^b^ ± 0.99

Groups of treatments D—divided application, S—single application; Bl—blend; opu—oil-based polyurethane polymer, o—oil-based polymer. The same letters next to the numbers describe no statistically significant differences between the treatments (Fisher’s LSD test, *p* ≤ 0.05). Each group of the treatment (divided, single, blend) was evaluated separately. The values represent the mean (*n* = 4) ± standard deviation (SD).

**Table 4:** Agrochemical properties of used soil.

Soil Parameter	Value	Devices	Ref.
Soil type	Stagnic Fluvisols (FL-st)		[59]
Clay	53%	Pipette apparatus (NEN 5753:2018)	[60]
pH (CaCl_2_)	6.6	pH meter, inoLab pH/ION Level 2 with SenTix 62 pH electrode	[61]
Soil electrical conductivity (EC)	0.05 mS/cm	EC meter, inolab pH/ION Level 2 with WTW TetraCon 325	[61]
Soil oxidizable carbon (C_ox_)	1.28%	Walkley-Black method	[62]
NH_4_^+^ (K_2_SO_4_)	1.48 mg/kg	UV/VIS Spectrometer, Unicam 8625	[61]
NO_3_^−^ (K_2_SO_4_)	17.2 mg/kg	NO_3_^−^-ISE	[61]
P (Mehlich III)	201 mg/kg	UV/VIS Spectrometer, Unicam 8625	[61]
K (Mehlich III)	367 mg/kg	AAS, ContrAA 700	[61]
Ca (Mehlich III)	3015 mg/kg	AAS, ContrAA 700	[61]
Mg (Mehlich III)	294 mg/kg	AAS, ContrAA 700	[61]
S (water-soluble)	13.8 mg/kg	ICP-MS, Agilent 7900	[61]

Mehlich III—soil test extractant.

**Table 5:** Design of treatments and nitrogen applications.

Treatment	Fertilizer	Application/Ratio	Dose of N in g per Pot (mg/kg Soil)		
			1st Fertilization	2nd Fertilization	Total Dose of N
D	CAN + CAN	divided (1:2)	0.408 (68)	0.848 (141)	1.256 (209)
D-opu	CAN + opu-CAN	divided (1:2)	0.408 (68)	0.848 (141)	1.256 (209)
D-o	CAN + o-CAN	divided (1:2)	0.408 (68)	0.848 (141)	1.256 (209)
S	CAN	single	1.256 (209)	-	1.256 (209)
S-opu	opu-CAN	single	1.256 (209)	-	1.256 (209)
S-o	o-CAN	single	1.256 (209)	-	1.256 (209)
Bl-opu	CAN + opu-CAN	single (blend 1:2)	0.408 + 0.848 (68 + 141)	-	1.256 (209)
Bl-o	CAN + o-CAN	single (blend 1:2)	0.408 + 0.848 (68 + 141)	-	1.256 (209)

Groups of treatments D—divided application, S—single application; Bl—blend; opu—oil-based polyurethane polymer, o—oil-based polymer.

**Table 6:** Experimental phases and dates.

Experimental Phases	Date	Rape Growth Stages	Term
Start of the experiment (sowing)	1 November 2018	seed Dry	
1st Fertilization	11 March 2019	nine or more leaves unfolded	
1st Plant and soil sampling 2nd Fertilization	2 April 2019	stem elongation	*t1*
2nd Plant and soil sampling	16 April 2019	flower bud emergence	*t2*
3rd Soil sampling; harvest	16 July 2019	harvested product	*t3*

## Data Availability

The data presented in this study are available on request from the corresponding author.
